# Cyclotron-Based Production of ^67^Cu for Radionuclide Theranostics via the ^70^Zn(p,α)^67^Cu Reaction

**DOI:** 10.3390/ph16020314

**Published:** 2023-02-17

**Authors:** Santiago Andrés Brühlmann, Martin Walther, Martin Kreller, Falco Reissig, Hans-Jürgen Pietzsch, Torsten Kniess, Klaus Kopka

**Affiliations:** 1 Helmholtz-Zentrum Dresden-Rossendorf, Institute of Radiopharmaceutical Cancer Research, Bautzner Landstraße 400, 01328 Dresden, Germany; 2 School of Science, Faculty of Chemistry and Food Chemistry, Technische Universität Dresden, Bergstraße 66, 01069 Dresden, Germany; 3 National Center for Tumor Diseases (NCT) Dresden, University Hospital Carl Gustav Carus, Fetscherstraße 74, 01307 Dresden, Germany; 4 German Cancer Consortium (DKTK), Partner Site Dresden, Fetscherstraße 74, 01307 Dresden, Germany

**Keywords:** copper-67, targetry, target chemistry, theranostics

## Abstract

Theranostic matched pairs of radionuclides have aroused interest during the last couple of years, and in that sense, copper is one element that has a lot to offer, and although ^61^Cu and ^64^Cu are slowly being established as diagnostic radionuclides for PET, the availability of the therapeutic counterpart ^67^Cu plays a key role for further radiopharmaceutical development in the future. Until now, the ^67^Cu shortage has not been solved; however, different production routes are being explored. This project aims at the production of no-carrier-added ^67^Cu with high radionuclidic purity with a medical 30MeV compact cyclotron via the ^70^Zn(p,α)^67^Cu reaction. With this purpose, proton irradiation of electrodeposited ^70^Zn targets was performed followed by two-step radiochemical separation based on solid-phase extraction. Activities of up to 600MBq ^67^Cu at end of bombardment, with radionuclidic purities over 99.5% and apparent molar activities of up to 80MBq/nmol, were quantified.

## 1. Introduction

Copper-67 (${}^{67}$Cu) is a pure $\beta^{-}$-emitter with a mean energy emission of 141 keV, a half-life of 61.83 h and γ-lines suitable for single-photon emission computed tomography (SPECT) imaging [1]. This radionuclide, cataloged as a low energy $\beta^{-}$-emitter similar to ${}^{177}$Lu, with a penetration of only 2.1 mm in soft tissue (CSDA approximation), is perfectly suitable for targeted endoradiotherapy [2,3]. Furthermore, the physical half-life makes it attractive for the tracking of slow pharmacokinetics of tracer molecules with different molecular weights, e.g., monoclonal antibodies (mAbs) [4,5]. Several studies have shown promising results with ${}^{67}$Cu radioconjugates especially in peptide receptor radionuclide therapy, with a few GBq${}^{67}$Cu [6,7,8]. Regarding the γ-lines suitable for SPECT imaging, the most prominent lines are 91 keV (7.0%), 93 keV (16%), and 184 keV (49%), with the last one being the most interesting for scintigraphic detection. It is also remarkable that ${}^{67}$Cu has no higher energy γ-emission (highest energy 394 keV with only 0.22% intensity) [1].

One main advantage of ${}^{67}$Cu is that it has not one but two diagnostic radionuclide counterparts. On the one hand ${}^{61}$Cu has a half-life of 3.339 h and average $\beta^{+}$ energy of 524 keV (51.6%) [9]. On the other hand, the widely studied ${}^{64}$Cu has a half-life of 12.70 h and average $\beta^{+}$ energy of 278 keV (17.6%) [9]. Both of these radionuclides can be obtained through proton irradiation with a low proton energy compact cyclotron and have been already produced in our group, with a weekly routine ${}^{64}$Cu production [10,11].

Diverse nuclear reactions were described in the past for the production of ${}^{67}$Cu. These production routes include fast neutron reactions on ${}^{67}$Zn [12,13], spallation of As and RbBr [14,15,16], cyclotron-based irradiation with protons on enriched ${}^{68}$Zn targets [17,18,19,20,21,22,23,24,25,26] and enriched ${}^{70}$Zn targets [21,27], reactions with deuterons on ${}^{70}$Zn and the photonuclear reaction on a massive ${}^{68}$Zn target [28,29,30]. During the last couple of years, the tendency has shifted to the production based on the ${}^{68}$Zn(γ,p)${}^{67}$Cu reaction with even research-grade ${}^{67}$Cu being commercially available offered by the company Iotron Medical [31]. However, it is still discussed whether the deuteron-based reaction can actually be the best option for the production of this radionuclide [3,32]. On the other hand, the ${}^{70}$Zn(p,α)${}^{67}$Cu reaction offers a low-energy alternative that is suitable for medical cyclotrons without significant co-production of undesired radiocopper nuclides.

The proton-induced reactions for the production of ${}^{67}$Cu are based on irradiation of enriched ${}^{68}$Zn or ${}^{70}$Zn targets. It is possible to avoid the production of byproducts through careful selection of a monoisotopical target material and the energy range. The ${}^{68}$Zn targets require higher proton energies than the ${}^{70}$Zn. Furthermore, at the same high energies (45 MeV to 70 MeV), the latter has a cross-section almost double the value of the former [26,33]. On the one hand, the ${}^{70}$Zn is more rare than the ${}^{68}$Zn (abundance of 0.61% and 18.45%, respectively) and thus more expensive. On the other hand, by low proton energy irradiation (<30 MeV) of a ${}^{70}$Zn target, production of the desired ${}^{67}$Cu avoiding the co-production of other copper (radio)isotopes is possible (e.g., $\beta^{+}$-emitter ${}^{64}$Cu and stable ${}^{65}$Cu), thus reaching a higher radionuclidic purity (RNP) as well as a higher molar activity [5,34,35].

The first available data about the cross-section of the ${}^{70}$Zn(p,α)${}^{67}$Cu reaction were given in a report by Levkovskii in the year 1991 [21], although no details of the method were given. Later on, Jamriska et al. [36] and Kastleiner et al. [27] showed consistent results. With a rather low cross-section (15 mb at max), only ${}^{67}$Cu activities of up to 700 MBq have been reported through this reaction so far [37].

We report here on the production of a ${}^{67}$Cu with high RNP via the ${}^{70}$Zn(p,α)${}^{67}$Cu reaction, from proton irradiation of an enriched ${}^{70}$Zn electrodeposited target with a 30 MeV compact cyclotron. Furthermore, we present the radiochemical separation and radiolabeling tests performed with the product [${}^{67}$Cu]CuCl${}_{2}$ solution.

## 2. Results and Discussion

### 2.1. Zinc Electrodeposition

**Figure 1:**
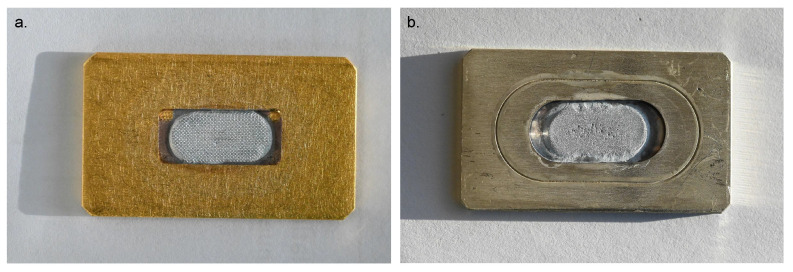
Representative electrodeposited ${}^{70}$Zn targets. (**a**) A 115 mg/cm^2^ target on a gold backing, from fresh ${}^{70}$Zn metal. (**b**) A 106 mg/cm^2^ target on a silver backing, from recycled [${}^{70}$Zn]ZnO.

Thick, dense, silver [${}^{70}$Zn]Zn targets were obtained after the electrodeposition, with masses between 115 mg to 160 mg representing area densities between 100 mg/cm^2^ and 140 mg/cm^2^ (i.a. target area ca. 116 mm^2^). These target thicknesses were used for the activation simulations verifying that satisfactory ${}^{67}$Cu yields could be reached. In some of these electrodeposited layers, some little holes were observed. As well, no major differences were found between electrodepositions from solutions containing fresh and recycled [${}^{70}$Zn]ZnSO${}_{4}$. In Figure 1, two typical ${}^{70}$Zn targets are shown, one corresponding to a fresh material electrodeposition on a gold backing and the other to a recovered material on a silver backing.

Electrodeposition efficiencies of up to 90% were achieved (defined as the ratio of deposited and initially loaded masses); however, in such cases the quality of the electrodeposition decreased. It was seen that lower [${}^{70}$Zn]Zn${}^{2+}$ concentrations produced uneven and defective targets, hence, electrodepositions with efficiencies ranging between 50% and 60% were preferred to prioritize the quality. Furthermore, the remaining solution containing [${}^{70}$Zn]Zn${}^{2+}$ could be reutilized for the next electrodeposition.

### 2.2. Target Parameter Calculations

**Figure 2:**
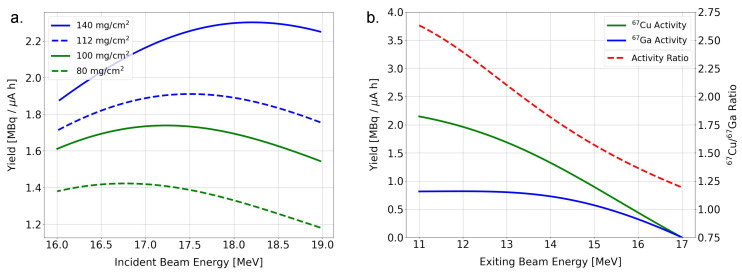
Results of the performed simulations. (**a**) ${}^{67}$Cu yield of the designed targets as a function of the energy of the incident beam. (**b**) Activity yields of ${}^{67}$Cu and ${}^{67}$Ga with their corresponding ratio.

Optimization of the incident proton beam energy was performed for the specified area densities. Subsequently, the theoretical ${}^{67}$Cu to ${}^{67}$Ga yield ratio for a fixed incident proton energy was also calculated. The result of the former simulation provided a proton energy that could be used for the irradiations. On the other hand, the latter simulation serves as a validation to estimate the degraded energy in the target. In Figure 2, simulation results are shown.

Based on these results, the first target irradiations were carried out with a proton energy of 17.5 MeV. However, the ${}^{67}$Cu yield and the ${}^{67}$Cu to ${}^{67}$Ga ratio were much lower than expected, which could be explained by uniformity in the electrodeposition and other minor factors. Nevertheless, both of these challenges could be overcome by reducing the energy of the proton beam and performing the following irradiations with an incident proton beam of 17.0 MeV.

Furthermore, thermal simulations were performed. Although the temperature profile in the target cannot be measured during or after irradiation, it was possible to validate the simulation with some targets showing partial melting. From the simulations, a current of 60 μA on a target with a gold backing could lead to temperatures close to the melting point of zinc. In this case, the thermal contact between the gold backing and the electrodeposited ${}^{70}$Zn played a key role. The explanation of why some targets, visually identical, suffered partial melting whereas others did not, lies in this resistive layer and was validated by the simulations. Moreover, the results with silver backing targets also matched the simulations, showing a greater endurance to higher currents due to the higher thermal conduction and lower stopping power for protons of silver in contrast to gold. In particular, a current of 60 μA on a target with a gold backing could lead to temperatures between 310 °C and 430 °C, whereas with a silver backing this temperature could reach up to 270 °C and 390 °C depending on the mentioned resistive layer. Considering that the melting point of zinc is 419.5 °C [38], the former targets are more likely to suffer from melting than the latter.

### 2.3. Target Irradiation

A first set of electrodeposited ${}^{70}$Zn targets on gold backings, 130 mg to 150 mg, were irradiated with an incident proton energy of 17.5 MeV and beam currents of 40 μA to 60 μA. The proton beam profile used for these irradiations was rather concentrated and some targets suffered partial melting. The ${}^{67}$Cu yield determined for these targets was quite variable, ranging from 0.2 MBq/(μA h) to 1.0 MBq/(μA h). As well, inconsistent ${}^{67}$Cu to ${}^{67}$Ga ratios (<1.0) were found.

Following these results, it became clear that an important part of the ${}^{67}$Cu activity was staying in the gold backing after target dissolution with HCl. This effect has already been described [27], however not in this magnitude, which amounted for up to 80% of the ${}^{67}$Cu activity.

Next, irradiations were performed with lower proton energy (17.0 MeV) and optimized broaded beam profile. The integrity of gold backing targets could not be assured for beam currents of over 50 μA in this case. In addition, the ${}^{67}$Cu retention on the gold backing was also present. Consequently, the remaining activity in the backing had to be recovered by dissolving the surface of the gold with a diluted Aqua Regia solution ( 6 M HCl/6 M HNO${}_{3}$ 3:1). Targets with silver backings were successfully irradiated with up to 60 μA beam currents and showed that longer HCl dissolution times or higher temperatures could remove more than 85% of the ${}^{67}$Cu activity.

The results of these irradiations were more consistent than the first set and exhibited a yield close to 1.0 MBq/(μA h) for a 130 mg target. The theoretical yield for such a target amounted to 1.75 MBq/(μA h) and 1.45 MBq/(μA h) when considering a 20% thickness reduction corresponding to 57% or 69% of the theoretical yields, respectively.

Activities of up to 600 MBq${}^{67}$Cu at end of bombardment (EOB) for a 140 mg${}^{70}$Zn on a silver backing were achieved with a beam current of 60 μA and an irradiation time of 12 h (2 days, 6 h, decay corrected effective 10.8 h). Silver backing targets showed no damage and could be further used for establishing a routine ${}^{67}$Cu production at the HZDR. Not least of all, considering the half-life of ${}^{67}$Cu, longer irradiations could also be performed to further increase the activities reached.

### 2.4. Radiochemical Separation and Product Characterization

Radiochemical separation of the ${}^{67}$Cu was performed with a two-step solid-phase extraction, consisting of a CU resin and a TK400 cartridge. The ${}^{67}$Cu activity was eluted from both columns with 8 M HCl, a necessary adjustment of the solution to a media suitable for radiolabeling. This last step was carried out by drying the product solution and redissolving it in water. Alternatively, the acidic solution was loaded onto a TK201 cartridge.

**Figure 3:**
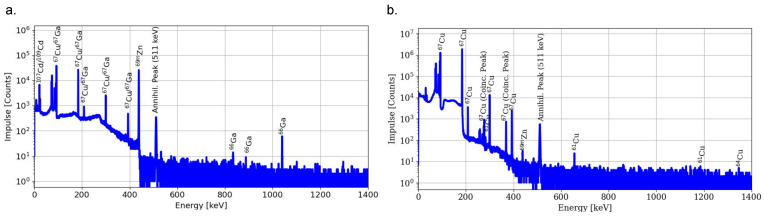
Gamma-ray spectra of raw target solution (**a**) and ${}^{67}$Cu product fraction (**b**).

The target was worked up 16 to 48 h after EOB to reduce the activity of short-lived radionuclides, e.g., ${}^{68}$Ga. The elution from the CU resin column contained up to 97.5% of the ${}^{67}$Cu activity, whereas 95% were obtained after the second step. Radiogallium impurities were detected in the raw target solution and product fraction, but in the latter only an estimation was possible. In Figure 3, the gamma-spectroscopy of the raw solution as well as of the product fraction is shown.

At the end of purification (EOP), the RNP of the ${}^{67}$Cu fraction was over 99.5%. The presence of ${}^{61}$Cu and ${}^{64}$Cu can be explained by the ${}^{64}$Zn(p,α)${}^{61}$Cu and ${}^{67}$Zn(p,α)${}^{64}$Cu reactions inherent to the low content of other zinc isotopes in the target material, but their contribution would be dropping with time due to their shorter half-lives. On the other hand, ${}^{67}$Ga content was estimated from the ${}^{66}$Ga activity since ${}^{67}$Ga shares the ${}^{67}$Cu γ-lines and was not possible to quantify at these low contents. Radionuclide impurities detected with its activity contribution are shown in Table 1.

Moreover, an apparent molar activity (AMA) of up to 80 MBq/nmol at EOB for 1,4,8,11-Tetraazacyclotetradecane-1,4,8,11-tetraacetic acid (TETA)-formed ${}^{67}$Cu complexes was quantified. Such AMA is satisfactory to further perform in vitro and in vivo experiments. It is also interesting to mention that the product obtained from recycled targets showed higher AMAs, which can be explained by further material purification during the recovery.

### 2.5. Recovery of Enriched ${}^{70}$Zn

By the workup of the target through precipitation of Zn(OH)${}_{2}$, a ${}^{70}$Zn recovery yield of over 92% was achieved. Higher [${}^{70}$Zn]Zn${}^{2+}$ concentrations were preferred in order to increase the recovery yield, i.e., two targets would be recovered together. As well, consistent precipitation was achieved within a pH range of 7 to 10. Another important aspect during the recovery is the speed and centrifugation time. When applying 7000 rpm (i.e., 5730 rcf) for three min, no apparent loss of the precipitate was seen, but reducing speed or time could decrease the recovery yield. Last but not least, the recycled [${}^{70}$Zn]ZnO was successfully used for further electrodepositions, thus closing the cycle and reducing the ${}^{67}$Cu production costs.

## 3. Materials and Methods

### 3.1. Reagents and Materials

The acid solutions were prepared with milli-Q water and ultrapure 30% hydrochloric acid (Merck KGaA), ultrapure 95% sulfuric acid (Roth GmbH), and ultrapure 69% nitric acid (Roth GmbH). For the electrodeposition, ultrapure 20% ammonia (Roth GmbH) was used. On the other hand, suprapure sodium hydroxide monohydrate (Merck KGaA), suprapure sodium acetate (Merck KGaA), and suprapure sodium chloride (Merck KGaA) were dissolved in milli-Q water. Triskem CU resin, TK400 1.0 mL, and TK201 1.0 mL cartridges were used.

High purity, 2 mm thick gold and silver foils were used as substrate for the electrodepositions.

Enriched ${}^{70}$Zn in the form of metallic powder was bought from ECP Rosatom Corporation Company, with isotopic composition (provider specifications) as shown in Table 2.

### 3.2. Zinc-70 Electrodeposition

Thick target zinc electrodeposition has been widely studied for the production of the $\beta^{+}$-emitter ${}^{68}$Ga [39,40] and copper radionuclides, such as ${}^{61}$Cu [11] and ${}^{67}$Cu [27,41]. In particular, thick target zinc electrodeposition is the most widely used method for the cyclotron-based production of ${}^{67}$Cu; results from enriched ${}^{68}$Zn [18,22,34] and ${}^{70}$Zn [27,36] targets have been reported. These electrodepositons were performed from diverse acid solutions, mainly based on hydrochloric [27,41] and sulfuric acid [34]. As well, different additives have been used to increase the electrochemical efficiency or enhance the quality of the deposited material, i.e., electrolytes and surfactants. One advantage of carrying out the electrodeposition of the targets is the possibility of recycling the expensive enriched ${}^{68}$Zn or ${}^{70}$Zn material after irradiation, closing the loop for the ${}^{67}$Cu production and thereby reducing costs.

Enriched ${}^{70}$Zn electrodeposition was carried out on gold and silver backings. These materials were chosen due to low proton activation, good thermal conduction and thus good target cooling, and being resistant to concentrated HCl solutions used for the target dissolution. Rectangular (24 mm × 40 mm) plates, with 0.5 mm deepening and an effective oval area of the electrodeposition close to 116 mm were used. Previous to the deposition, the backings were washed with concentrated HCl to guarantee the absence of contaminants and then placed into the electrodeposition device, followed by a magnetic stirrer and the prepared solution. This device included a platinum cathode, whereas the target backing acted as the anode. The electrodepositions were carried out between four and five hours, with a fixed current of 35 mA and a voltage of (3.9 ± 0.2) V. The magnetic stirrer was used during the whole electrodeposition process, at a speed of 300 rpm.

After the first attempts at zinc electrodeposition from hydrochloric solutions with low-quality results, sulfuric solutions were studied. Starting with the conditions described by Sadeghi et al. [39], and after changing the acid media from HCl to H${}_{2}$SO${}_{4}$, several experiments were carried out, modifying the parameters in order to achieve the desired quality. The optimized parameters were as follows: Zn${}^{2+}$ concentration 10–20 g/L, (NH${}_{4}$)${}_{2}$SO${}_{4}$ 34–59 g/L, pH = 2, solution volume 15 mL to 20 mL. It is important to mention that the concentration and volume ranges provided some flexibility when electrodepositing from recovered targets and reusing electrodeposition solutions.

Preparation of the electrodeposition solution consisted of the dissolution of highly enriched ${}^{70}$Zn metal powder (97.5%) in diluted sulfuric acid 47.5%, followed by careful addition of milli-Q water and ammonia solution to increase the pH (pH ca. 2). On the other hand, the recovered enriched [${}^{70}$Zn]ZnO was also prepared in the same way.

### 3.3. Target Parameter Calculations

The activity obtained at the EOB after irradiation can be calculated by Equation (1) (A${}_{EOB}$ in Bq), where N${}_{A}$ stands for the Avogadro constant, A, for the mass number of the target in g/mol, I for the proton beam current in μA, $q_{e}$ is the electron charge in μC, E${}_{in}$ and E${}_{out}$ are the incident and exiting energy in MeV, σ is the cross-section of the reaction in cm^2^, S(E) is the stopping power of the material in MeV cm^2^/g, $t_{irr}$ is the irradiation time, and $T_{1/2}$ is the physical half-life of the radionuclide in the same units [42].

(1) $$A_{EOB}=\frac{N_{A}}{A}\;\cdot \;\frac{I}{q_{e}}\;\cdot \;\int_{E_{out}}^{E_{in}}\frac{\sigma (E)}{S(E)}dE\;\cdot \;\left(1-2^{-\;\frac{t_{irr}}{T_{1/2}}}\right)$$

On the other hand, the heat generation within the target can be described by the Equation (2), where q is the heating power in W, I the current in μA, and E${}_{in}$ and E${}_{out}$ are the incident and exiting energy of the ion beam in MeV.

(2) $$q=I\;\cdot \;(E_{in}-E_{out})$$

The main challenge of this heat production is the possible melting down of the target, and the consequent loss of the expensive material and contamination of the accelerator facility. Some authors have proposed optimization tools considering activity yields and heat generation; however, no standard procedure has been established [43,44]. One important aspect to also take into account is the lack of an integral analysis not only of the heat generation but also of the thermal properties of the materials involved as well as the cooling conditions of the system.

Hence, simulations were performed to optimize the ${}^{67}$Cu production. The effect of changing the incident proton energy used for the irradiation with a fixed target thickness was studied. Two borderline target thicknesses were analyzed based on the first electrodepositing results, corresponding to area densities of 100 mg/cm^2^ and 140 mg/cm^2^. Due to possible defects on the electrodeposition, e.g., electrodeposited material on borders, reductions of 20% on each area density were also considered. Although thicker targets are desired in order to increase the yield, due to the limitations of the cooling system and the target production method, no further studies of larger targets were performed. A self-developed Python program based on cross-section data provided by IAEA [45] and stopping power data from SRIM [46] was used for optimization purposes.

**Figure 4:**
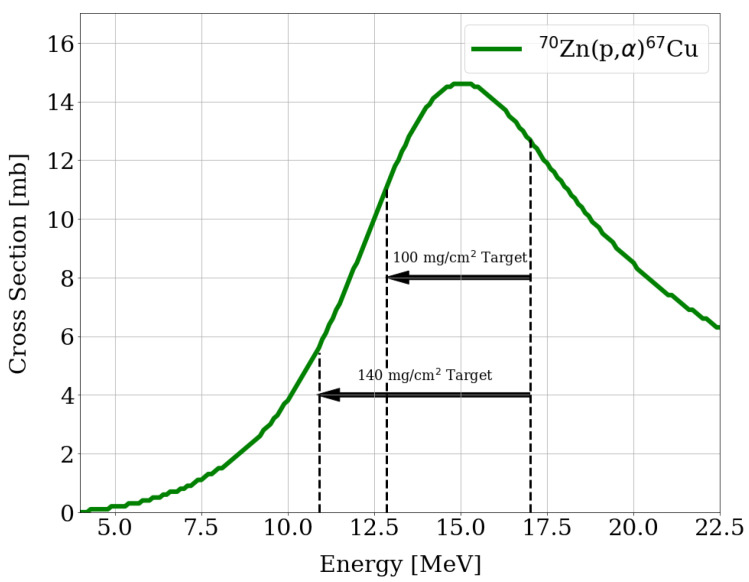
Cross-section of the ${}^{70}$Zn(p,α)${}^{67}$Cu reaction. In dotted lines, the degraded energy estimated in the target and used for the simulations are indicated.

The cross-section of the ${}^{70}$Zn(p,α)${}^{67}$Cu reaction is shown in Figure 4 along the estimated proton energy degraded, in the 100 mg/cm^2^ and 140 mg/cm^2^ targets, with a 30° tilted target [45]. As can be seen, there is a maximum in the cross-section at about 15 MeV; thus, the energy range used for the irradiation included this energy.

On the other hand, thermal simulations were performed to determine the maximal current that could be used for the designed target. Such simulations were performed with the COMSOL platform [47]. The thermal contact between the target and backing was simulated by including an equivalent resistive layer in the simulation. Evaluation of gold and silver as backing material was carried out.

### 3.4. Target Irradiation

The target irradiation was carried out at the TR-Flex (ACSI) cyclotron installed at the HZDR [48], with the 30° tilted target configuration in order to reduce the surface current density at the target. This tilted target has an apparent thickness of twice the real one.

Incident proton energies of 17.5 MeV, 17.0 MeV, and 16.8 MeV were used, with beam currents ranging from 30 μA to 60 μA. The proton beam profile was not measured for this specific experiment; however, the beam profile has been characterized previously with a FWHM of 12 mm to 14 mm in an energy range of 14 MeV to 30 MeV [48]. The FWHM was expected to be below 10 mm for the concentrated ion beam.

Cooling of the target was performed with water on the backside (3 L/min, 25 °C) and with helium at the front (300 L/min, 25 °C). Irradiation times ranged between 4 and 6 h, with longest irradiation times of two times 6 h on consecutive days.

### 3.5. Radiochemical Separation and Product Characterization

**Figure 5:**
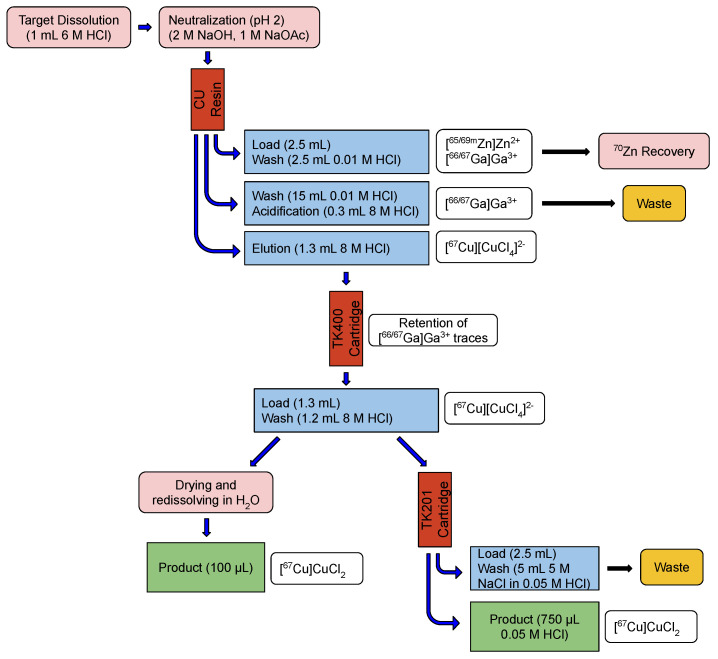
Simplified scheme of the radiochemical separation, consisting of two-step solid-phase extraction columns followed by adjustment of the high acidic [${}^{67}$Cu][CuCl${}_{4}$]${}^{2-}$ solution to media suitable for radiolabeling.

Radiochemical separation of the [${}^{67}$Cu]CuCl${}_{2}$ consisted of a two-step solid-phase extraction. After dissolution of the target in 1 mL 6 M HCl, the solution was neutralized by addition of 2 M NaOH, volume estimated by knowing the ${}^{70}$Zn target mass and acid volume. Fine-tuning of the pH to reach a value of 2 was performed with 1 M NaOAc. The resulting solution (2.0 to 2.5 mL) was loaded onto a preconditioned (3 × 1.0 mL 8 M HCl followed by 3 × 5.0 mL 0.01 M HCl) 1.0 mL CU resin column, similar to the work described by Thieme et al. [11]. After washing with 3 × 5.0 mL 0.01 M HCl, the ${}^{67}$Cu elution was carried out with 1.3 mL 8 M HCl. Since in this fraction ${}^{67}$Ga activities accounting up to 2% of total activity were detected, the use of a second column to retain the radiogallium was forced. With this purpose, a preconditioned (3 × 1.0 mL 0.01 M HCl followed by 3 × 1.0 mL 8 M HCl) 1.0 mL TK400 cartridge was used. After loading the elution from the first column, the cartridge was washed with 1.2 mL 8 M HCl and these two fractions collected. This product solution was dried on a rotation evaporator and redissolved in water to obtain the product. Alternatively, the 8 M HCl solution containing the [${}^{67}$Cu][CuCl${}_{4}$]${}^{2-}$ could be loaded onto a preconditioned (3 × 1.0 mL 0.01 M HCl followed by 3 × 1.0 mL 8 M HCl) 1.0 mL TK201 cartridge. In this case, the column was washed with 5 M NaCl in 0.05 M HCl like proposed by Svedjehed et al. [49] and the radiocopper eluted in 3 × 0.25 mL 0.05 M HCl. Either way, a ready-to-label solution was achieved. A simplified scheme of the separation is showed in Figure 5.

Characterizations of the target raw solution, CU resin, and TK400 cartridge elutions were performed by high-resolution gamma spectroscopy using an energy- and efficiency-calibrated Mirion Technologies (Canberra) CryoPulse 5 HPGe detector. From these fractions, 1.0 μL of activity (i.e., 100 kBq to 600 kBq) was added to a 200 μL tube with calibrated geometry for the gamma spectroscopy measurement. The time of measurement was set for 600 s live-time, with dead-time below 5%. A one-hour measurement of the product fraction was performed to better quantify impurities. Activities were automatically calculated with the software Genie2000 (V. 3.4.1).

On the other hand, the AMA of the product was quantified by titration with the macrocyclic ligand TETA. Radiolabeling followed the method proposed by Thieme et al. [10]. Basically, approximately 2 MBq [${}^{67}$Cu]CuCl${}_{2}$ was added to solutions containing different complexing agent concentrations (no buffer needed), and mixed for 30 min at room temperature. The TETA complex was performed on an iTLC-SA paper developed with 0.9% NaCl solution. In this case, [${}^{67}$Cu]CuCl${}_{2}$ remains at the start whereas the radiometal complex runs to the front. Complexation of over 90% was used to determine the AMA.

### 3.6. Target Recycling: Recovery of Enriched ${}^{70}$Zn

Among the five stable isotopes of zinc, ${}^{70}$Zn is that with the lowest abundance [45], with a direct consequence of an extremely high price for enriched ${}^{70}$Zn material. When considering that each electrodeposited target contains between 115 mg and 160 mg of highly enriched ${}^{70}$Zn, the importance of developing a recovery strategy for the irradiated target material becomes essential. The recycling of this target material can be performed through the precipitation of Zn(OH)${}_{2}$, followed by annealing to recover the oxide form [50].

After separation, the fraction containing [${}^{70}$Zn]ZnCl${}_{2}$ was left to decay for at least 45 days. Two zinc radionuclides have been detected in this fraction, short-lived ${}^{69m}$Zn, half-life 13.756 h, and long-lived ${}^{65}$Zn, half-life 244 day. Although shorter decay times would be enough to get rid of the former, the latter radionuclide is prompted to accumulate after irradiation of recovered targets [35]. On the other hand, the radionuclide ${}^{67}$Ga, half-life 3.26 day, is also present in this fraction, with activities comparable to that of ${}^{67}$Cu at EOB.

Precipitation of [${}^{70}$Zn]Zn(OH)${}_{2}$ was achieved by the addition of 6 M NaOH until a pH of around 8 was reached, with the lowest Zn(OH)${}_{2}$ solubility observed, based on the work of Katabuchi et al. [50]. The separation consisted of the centrifugation, separation of the supernatant and further washing.

Usually, for target recycling, two fractions containing [${}^{70}$Zn]ZnCl${}_{2}$ were combined, i.e., two targets, and the addition of 6 M NaOH would be carried out slowly in order to avoid having an excessively basic solution. After the pH was set to around 8, the separation was obtained through centrifugation of the solution. A speed of 7000 rpm during 3 min was used for this purpose. The supernatant was then removed and the precipitate was washed with water for a new centrifugation cycle. This washing/centrifugation process was repeated five times to assure the purity of the material and reduce the NaCl content. The obtained precipitate was then transferred into a quartz beaker where it was dried in a furnace at a temperature of 105 °C for 4 h, as proposed by Zueva et al. [51]. Then, the [${}^{70}$Zn]Zn(OH)${}_{2}$ precipitate could be redissolved in diluted H${}_{2}$SO${}_{4}$ for new electrodeposition or alternatively annealed in a hotplate at 450 °C for 3 h in order to get [${}^{70}$Zn]ZnO for storage.

## 4. Conclusions

To sum up, the methodology presented allowed the production of considerable amounts of no-carrier-added [${}^{67}$Cu]CuCl${}_{2}$ with high RNP and admissible AMA. Furthermore, a complete process for ${}^{67}$Cu production via the ${}^{70}$Zn(p,α)${}^{67}$Cu reaction with a compact cyclotron was provided, including the targetry, radiochemical separation, and target recycling. Moreover, RNP of over 99.5% ${}^{67}$Cu with 0.3% ${}^{64}$Cu as the main impurity were quantified at EOP. In addition, the determined AMAs of up to 80 MBq/nmol are appropriate for performing in vitro and in vivo studies. Considering that higher ${}^{67}$Cu yields could also be reached by longer irradiation of the described targets, the route proposed offers one alternative to the production of close to the GBq range activities of high purity [${}^{67}$Cu]CuCl${}_{2}$.

## Figures and Tables

**Table 1:** Radionuclide impurities detected at the ${}^{67}$Cu product fraction and its percentage of the total activity at EOP.

Radionuclide	${}^{61}$Cu	${}^{64}$Cu	${}^{66}$Ga	${}^{67}$Ga	${}^{69m}$Zn
Activity %	<0.03	<0.3	<0.05	<0.1	<0.003

**Table 2:** Isotopic composition of the ${}^{70}$Zn metallic powder used for the target electrodeposition.

${}^{64}$Zn	${}^{66}$Zn	${}^{67}$Zn	${}^{68}$Zn	${}^{70}$Zn
0.1%	0.1%	0.1%	2.2%	97.5%

## Data Availability

Not applicable.
